# Potential for using simulated altitude as a means of prehabilitation: a physiology study

**DOI:** 10.1111/anae.16158

**Published:** 2023-10-25

**Authors:** L. A. Brown, J. A. Griffiths, P. Santer, P. M. Jakeman, T. G. Smith

**Affiliations:** ^1^ Royal Melbourne Hospital Melbourne Australia; ^2^ Nuffield Department of Anaesthesia Oxford University Hospitals NHS Foundation Trust Oxford UK; ^3^ Department of Anesthesia, Critical Care and Pain Medicine Beth Israel Deaconess Medical Center, Harvard Medical School Boston MA USA; ^4^ Health Research Institute and Department of Physical Education and Sport Sciences University of Limerick Limerick Ireland; ^5^ Centre for Human and Applied Physiological Sciences King's College London London UK; ^6^ Department of Anaesthesia Guy's and St Thomas' NHS Foundation Trust London UK

**Keywords:** altitude training, haematological fitness, hypoxic conditioning, peri‐operative medicine, prehabilitation

## Abstract

The current pandemic of surgical complications necessitates urgent and pragmatic innovation to reduce postoperative morbidity and mortality, which are associated with poor pre‐operative fitness and anaemia. Exercise prehabilitation is a compelling strategy, but it has proven difficult to establish that it improves outcomes either in isolation or as part of a multimodal approach. Simulated altitude exposure improves performance in athletes and offers a novel potential means of improving cardiorespiratory and metabolic fitness and alleviating anaemia within the prehabilitation window. We aimed to provide an initial physiological foundation for ‘altitude prehabilitation’ by determining the physiological effects of one week of simulated altitude (F_I_O_2_ 15%, equivalent to approximately 2438 m (8000 ft)) in older sedentary volunteers. The study used a randomised, double‐blind, sham‐controlled crossover design. Eight participants spent counterbalanced normoxic and hypoxic weeks in a residential hypoxia facility and underwent repeated cardiopulmonary exercise tests. Mean (SD) age of participants was 64 (7) y and they were unfit, with mean (SD) baseline anaerobic threshold 12 (2) ml.kg^‐1^.min^‐1^ and mean (SD) peak V̇O_2_ 15 (3) ml.kg^‐1^.min^‐1^. Hypoxia was mild (mean (SD) S_p_O_2_ 93 (2) %, p < 0.001) and well‐tolerated. Despite some indication of greater peak exercise capacity following hypoxia, overall there was no effect of simulated altitude on anaerobic threshold or peak V̇O_2_. However, hypoxia induced a substantial increase in mean (SD) haemoglobin of 1.5 (2.7) g.dl^‐1^ (13% increase, p = 0.028). This study has established the concept and feasibility of ‘altitude prehabilitation’ and demonstrated specific potential for improving haematological fitness. Physiologically, there is value in exploring a possible role for simulated altitude in pre‐operative optimisation.

## Introduction

Postoperative complications are a major cause of morbidity and mortality, to the extent that they have been described as a ‘hidden pandemic’ that threatens the future sustainability of surgical care [1]. This is likely to worsen as the average age and comorbid complexity of patients requiring anaesthesia increases. Lower pre‐operative fitness measured using cardiopulmonary exercise testing (CPET) is associated with poorer postoperative outcomes, and CPET is used widely in peri‐operative medicine to stratify risk [2]. The possibility of improving outcomes by improving pre‐operative fitness is intuitively compelling, and many studies have investigated the use of exercise prehabilitation. While encouraging results have been reported, pre‐operative exercise programmes do not always improve cardiorespiratory function, and it has been difficult to demonstrate definitive evidence of clinical benefit either in isolation or in combination with other pre‐operative interventions (e.g. nutritional optimisation and psychological support [3, 4, 5, 6]).

Exercise prehabilitation can also be constrained by logistical factors such as the time available before surgery and access to suitable facilities, and some patients are unable to exercise due to comorbidities or disability. From an evolutionary perspective, our biological predisposition to minimise energy expenditure presents a more fundamental hurdle; it is intrinsically challenging to exercise people who are unfit because they avoid physical activity, and some patients are not interested in engaging.

Given the challenges and limitations of exercise prehabilitation, other potential means of improving fitness pre‐operatively should be considered and explored. The concept of altitude training presents one such alternative. Exposure to a hypoxic environment that simulates altitude could theoretically improve cardiorespiratory and metabolic fitness passively, and by stimulating erythropoiesis could also help to correct pre‐operative anaemia, which is common and associated with poorer patient outcomes [7, 8, 9, 10].

Exposure to hypoxia results in a suite of responses that generally work to improve the uptake, transfer and utilisation of oxygen [11]. The hypoxia‐inducible factor family of transcription factors play a central regulatory role in these homeostatic changes, both intracellularly and at the systemic level (e.g. increased ventilation, hypoxic pulmonary vasoconstriction), and even very mild hypoxia switches on hypoxia‐inducible factor and provokes downstream responses [11]. Erythropoietin is activated by hypoxia‐inducible factor and substantially increases within 4 h of take‐off in airline passengers [12], who are mildly hypoxic with S_p_O_2_ around 90–95% due to reduced cabin pressure [13]. Expressed as its altitude equivalent, cabin pressure altitude typically ranges between 1524 m (5000 ft) and 2438 m (8000 ft) on commercial airline flights and stimulates classic physiological responses in‐flight including ventilatory acclimatisation and hypoxic pulmonary vasoconstriction [14, 15, 16].

It is notable that mild‐to‐moderate hypoxia can be harmful or beneficial depending on circumstances and individual vulnerability. For example, in susceptible airline passengers and perhaps a minority of patients in the peri‐operative setting, mild hypoxia can trigger excessive pulmonary vascular responses that may contribute to in‐flight medical emergencies and peri‐operative complications, respectively [17, 18]. However, moderate altitudes (around 1524 m (8000 ft)) similar to aircraft cabin conditions can also be beneficial over the longer term. Mortality rates are lower in long‐term residents at these altitudes, and short‐term mild hypoxia can enhance cardiorespiratory and metabolic function [19]. Moderate altitudes are favoured for altitude training, which is a well‐established technique for improving performance in endurance athletes [20]. Even elite athletes can improve their performance with 2–3 weeks of altitude exposure, and relative benefits are greater when baseline fitness is lower [20, 21, 22]. Although increasing red cell mass is a desired outcome, non‐haematological factors such as increased mitochondrial efficiency are also important [23]. Mountaineers and soldiers also ‘pre‐acclimatise’ by staging for several days at similar elevations which protect against reduced cardiopulmonary performance during subsequent higher ascents [24].

Many athletes use simulated altitude exposure, also known as hypoxic conditioning, to achieve benefits passively without travelling to mountainous regions. Athletes following a simulated ‘live high/train low’ regimen from home use a hypoxic canopy while sleeping, which is ‘hypoxicated’ using molecular sieve technology to lower the F_I_O_2_, typically to 15% (equivalent to the P_I_O_2_ at an altitude of around 2438 m (8000 ft)).

Hypoxic conditioning has also been used as a medical therapy and, in limited studies of sedentary and older volunteers and patients, hypoxia spread intermittently over several weeks has been reported to improve the anaerobic threshold and other cardiopulmonary variables [25]. If this were to translate to the peri‐operative setting, simulated altitude exposure might offer an additional means of improving anaerobic threshold and other CPET indicators of increased peri‐operative risk. These include anaerobic threshold < 11 ml.kg^‐1^.min^‐1^ and peak V̇O_2_ < 15 ml.kg^‐1^.min^‐1^ [26], although we note that cardiopulmonary fitness clearly follows a spectrum, and bluntly applying binary cut‐offs is problematic.

If the physiological effects were sufficiently favourable, it is conceivable that a pre‐operative period of hypoxic conditioning, either as an inpatient or at home, would be both clinically beneficial and cost‐effective. This would require a level of hypoxia and duration of exposure that is sufficient to switch on hypoxia‐responsive genes and stimulate physiological adaptations, and that is also safe and short enough to be used in practice. Balancing these considerations, we aimed to determine the physiological effects of one week at a simulated altitude of 2438 m (8000 ft) in older sedentary volunteers.

## Methods

This physiology study took place at a residential hypoxia facility and used a randomised, double‐blind, sham‐controlled crossover design. It was approved by the Faculty of Education and Health Sciences Research Ethics Committee at the University of Limerick, where the experiments took place, and was conducted in accordance with the principles of the Declaration of Helsinki. Participants provided written informed consent and were financially reimbursed for their time.

Participants were required to be aged 50–70 y and generally healthy but with a sedentary lifestyle involving no structured exercise and only minimal physical activity. Volunteers were recruited locally through advertisements targeted particularly at bridge clubs. As the protocol involved exposure to simulated altitudes similar to those experienced in a commercial aircraft cabin, potential participants were screened to exclude serious medical conditions that would preclude taking an airline flight [13]. They were also screened to exclude a recent history of residence at high altitude.

The study was undertaken at the National Altitude Training Centre, University of Limerick (located at sea level). This residential hypoxia facility, or ‘hypoxic house’, is designed for prolonged stays and has a total living area of 122 m^2^ including seven hotel‐style bedrooms over two floors and a communal living area and kitchen. A hypoxic air generator (Hypoxico K2‐3000, Hypoxico Inc, New York, NY, USA) separates compressed air into an oxygen‐enriched stream, which is discarded, and a nitrogen‐enriched (i.e. oxygen‐deplete) stream that is piped throughout the house. The system is intended to achieve mild‐to‐moderate reductions in F_I_O_2_ for the purposes of altitude training and is not capable of generating extreme hypoxia. The F_I_O_2_ can be controlled independently in individual rooms by selecting the desired target value on a control unit located in a small control room inside the building that is only accessible to staff. This control unit receives input from oxygen sensors in each room and, via a feedback control system, regulates the supply of hypoxic air to each individual room to maintain the target F_I_O_2_. A single F_I_O_2_ value can be selected for the whole house, as it was for this study. Oxygen and carbon dioxide are monitored continuously throughout the building by alarmed sensors, but the house is not airtight or specially sealed in any way, and there is a constant flow of air with natural leakage from the building so that, as for any regular residential building, there is no accumulation of expired carbon dioxide.

An exposure duration of one week was chosen following careful consideration of the literature and consultation with experts across the field. Participants resided at the National Altitude Training Centre for seven days on two occasions: a ‘normoxic week’ and a ‘hypoxic week’. The order of the exposures was counterbalanced after block randomising participants into two equal groups, such that half the participants experienced normoxia first and half experienced hypoxia first. Experimental exposures were separated by two weeks to avoid the potential for physiological effects from one exposure to confound the other, and participants were blinded to the order of exposures. During the normoxic week, compressed air was intermittently piped throughout the house so that the audible experience was similar across both weeks. An unblinded member of the research team resided in the facility during all exposures and monitored the environmental conditions and participants throughout, ensuring that any potential concerns could be addressed immediately and that the study protocol was followed correctly.

An F_I_O_2_ of 15% was chosen for the hypoxic week, as the equivalent altitude of around 2438 m (8000 ft) is used successfully for altitude training and is generally safe for older people [13, 27]. To comply with the facility's standard operating procedures, the hypoxic exposure was induced incrementally over the first two days, with stepwise decreases and intermittent increases in oxygen achieving a mean F_I_O_2_ of 17% on day 1 and 16% on day 2. From day 3 onwards, participants were exposed to a constant F_I_O_2_ of 15% and did not leave the house until the end of the study week, when they exited at 08.00 on day 8. Meals were prepared by a professional chef with oversight by a dietician to achieve similar caloric intake across all study weeks. Participants did not undertake any exercise while residing in the house. They had access to seated activities such as reading and television; they spent much of their time playing card games. Between residential exposures, participants returned to their normal sedentary lifestyles, which did not involve any exercise. They did not travel beyond the local area in the lead‐up to the study or during the period between exposures.

Participants underwent a familiarisation CPET at the time of recruitment and then underwent repeat tests immediately before and after each week‐long exposure. The tests were performed by the same trained investigator throughout the study and followed a standard peri‐operative CPET protocol [2] using an Ultima CardiO2 system and BreezeSuite software (MGC Diagnostics, Saint Paul, MN, USA) with a Lode cycle (Lode BV, Groningen, the Netherlands). Ramp protocol, seat height and handlebar height were tailored to each individual participant and did not change throughout the study. Test results were reported blind by a consultant anaesthetist experienced in peri‐operative CPET.

Spirometry, iron studies and body fat percentage via DEXA scan (GE Lunar iDXA, GE Healthcare Lunar, Madison, WI, USA) were undertaken at baseline. Haemoglobin and erythropoietin (Quantikine IVD human EPO ELISA, R&D Systems, Abingdon, UK) were measured at baseline, on day 4 in the house and immediately following each week‐long exposure. During residential exposures, ambient F_I_O_2_ and F_I_CO_2_ were monitored continuously via the environmental control system and manually by the resident researcher, who ensured that the desired F_I_O_2_ was maintained. Vital signs were recorded at the same time each morning, including heart rate, respiratory rate and oxygen saturation. Participants were advised to report any symptoms immediately and were formally assessed with a daily Lake Louise questionnaire for symptoms of acute mountain sickness [28].

The primary outcome measure was the change in anaerobic threshold after one week of hypoxia. Secondary outcomes were the changes in other CPET variables (including peak V̇O_2_) and haematological variables (haemoglobin and erythropoietin). We aimed to study eight participants, which is typical for physiology studies generally and specifically when investigating systemic effects of hypoxia [12, 14, 15, 29]. This was also consistent with a sample size calculation based on a minimum difference for anaerobic threshold (1.5 ml.kg^‐1^.min^‐1^) and SD (1.1 ml.kg^‐1^.min^‐1^) used in analogous work [30] (power of 80% and two‐sided α of 0.05).

We performed statistical analysis using SPSS 24.0 (IBM, Armonk, NY, USA) and GraphPad Prism 7.0a (GraphPad Software, La Jolla, CA, USA). Student's paired t‐tests were used to compare differences in means, and longitudinal analysis of the effects of different exposures was performed by linear mixed effects modelling which included the factors exposure and day as well as their interaction term as fixed effects. A p value < 0.05 was considered statistically significant.

## Results

Baseline characteristics of participants are shown in Table 1. Eight participants completed the study and broadly reflected a population that may require prehabilitation: on average participants were aged 64 y with raised BMI, high body fat percentage, anaemia, impaired spirometry and baseline mean (SD) anaerobic threshold (12 (2) ml.kg^‐1^.min^‐1^) and mean (SD) peak V̇O_2_ (15 (3) ml.kg^‐1^.min^‐1^) in the ‘unfit’ range.

**Table 1:** Baseline characteristics of participants (unit (reference range)). Values are mean (SD).

	All participants
	n = 8
Age; y	64 (7)
Sex; female	5
Height; m	1.68 (0.08)
Weight; kg	77 (16)
BMI; kg.m^‐2^	27 (4)
Body fat percentage; %	40 (6)
S_p_O_2_; %	96 (2)
Heart rate; bpm	80 (13)
Blood pressure; mmHg	
Systolic	127 (16)
Diastolic	71 (8)
FEV_1_; l	2.11 (0.83)
FEV_1_; % of predicted	76 (26)
FVC; l	2.96 (0.94)
FVC; % of predicted	80 (17)
FEV_1_/FVC ratio; %	72 (19)
Haemoglobin; g.dl^‐1^ (11.5–18.0 g.dl^‐1^)	11.0 (1.5)
Haematocrit; % (34.9–50.0%)	32 (4)
Ferritin; μg.l^‐1^ (20–300 μg. l^‐1^)	136 (116)
Transferrin; g l^‐1^ (1.74–3.64)	2.63 (0.35)
Transferrin saturation; % (15–45%)	24 (8)
Serum iron; μmol. l^‐1^ (11.6–31.3)	14 (5)
Anaerobic threshold; ml.kg^‐1^.min^‐1^	12 (2)
Peak V̇O_2_; ml.kg^‐1^.min^‐1^	15 (3)
V̇_E_/V̇CO_2_ at anaerobic threshold	28 (5)

FEV_1_, forced expiratory volume in the first second; FVC, forced vital capacity; V̇_E_/V̇CO_2_, ventilatory equivalent for CO_2_. Where normal ranges vary with sex, the widest range is given. Cardiopulmonary exercise test data are from the initial familiarisation CPET.

**Figure 1:**
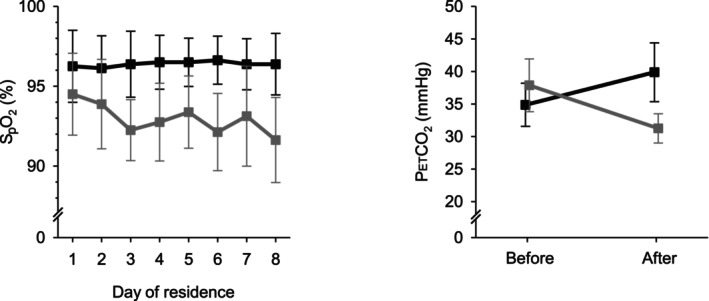
Effect of residential exposure on oxygenation and end‐tidal carbon dioxide. S_p_O_2_ and end‐tidal partial pressure of carbon dioxide (P_ET_CO_2_) are shown. Black, normoxic week; grey, hypoxic week. Error bars indicate SD.

Figure 1 shows S_p_O_2_ during the residential exposures and confirms that a fall in oxygenation was achieved. Hypoxia resulted in a significant fall in mean (SD) S_p_O_2_ from 96 (2) % during the normoxic week to 93 (2) % during the hypoxic week (p < 0.001). The lowest recorded S_p_O_2_ for each participant ranged from 86% to 93%. Figure 1 also shows the fall in end‐tidal partial pressure of carbon dioxide associated with simulated altitude exposure, reflecting classic ventilatory acclimatisation to hypoxia [11, 29] and physiologically confirming that an effective hypoxic stimulus had been applied. Heart rate was slightly higher during the hypoxic week than the normoxic week (mean (SD) 77 (13) vs. 84 (4) bpm, p = 0.002), and there was also a very small but statistically significant increase in the respiratory rate in response to hypoxia (mean (SD) 16 (2) vs. 17 (2) breaths.min^‐1^, p < 0.001). Hypoxic exposures were well‐tolerated and no participant developed acute mountain sickness.

**Figure 2:**
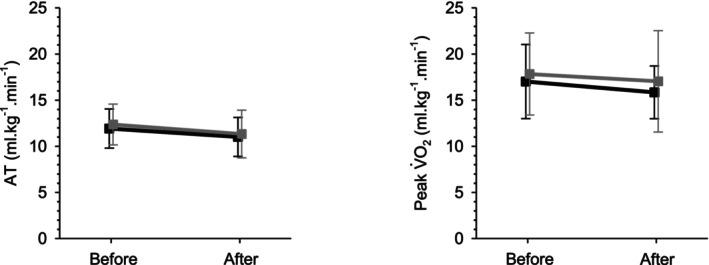
Main cardiopulmonary exercise test outcomes. Anaerobic threshold (AT) and peak V̇O_2_ are shown before and after residential exposures. Black, normoxic week; grey, hypoxic week. Error bars indicate SD.

Figure 2 shows anaerobic threshold and peak V̇O_2_ before and after each week in the facility. Table 2 shows further CPET variables. There was no effect of simulated altitude exposure on anaerobic threshold. Peak V̇O_2_ was likewise unaffected by residence in the facility or by hypoxia, although some peak variables were increased at the end of the hypoxic week compared with the beginning, including a 31 s increase in the duration of test (5% increase, p = 0.023), a 12 W increase in peak work rate (11% increase, p = 0.034) and a 17 bpm increase in peak heart rate (15% increase, p = 0.040). However, none of these differences were statistically significant when compared with the normoxic control week data. The ventilatory equivalent for carbon dioxide (V̇_E_/V̇CO_2_) at anaerobic threshold was increased following exposure to hypoxia (p < 0.001), although a similar change has been reported acutely at around 2438 m (8000 ft) and this finding is difficult to interpret in the context of ventilatory acclimatisation to hypoxia [31].

**Table 2:** Cardiopulmonary exercise testing data before and after exposure to normoxia and hypoxia. Values are mean (SD).

	Normoxia				Hypoxia				p value (normoxia vs. hypoxia)
	Pre exposure	Post exposure	Difference (pre‐post)	p value (pre‐post)	Pre exposure	Post exposure	Difference (pre‐post)	p value (pre‐post)	
**Baseline data**									
Ventilation; l.min^‐1^	8.5 (3.0)	9.0 (2.8)	0.5	0.354	8.5 (2.1)	10.2 (1.7)	1.8	0.007	0.184
**Data at anaerobic threshold**									
V̇O_2_; ml.kg^‐1^.min^‐1^	11.9 (2.1)	11.0 (2.1)	‐0.9	0.202	12.4 (2.2)	11.3 (2.6)	‐1.0	0.289	0.914
V̇O_2_; ml.min^‐1^	921 (241)	875 (279)	‐46	0.426	959 (282)	895 (310)	‐64	0.350	0.844
V̇_E_/V̇CO_2_	31 (5)	27 (4)	‐4	0.001	29 (5)	33 (4)	4	< 0.001	< 0.001
Work rate; W	66 (25)	66 (23)	0	0.955	66 (21)	68 (23)	2	0.656	0.702
**Data at peak V̇O**_**2**_									
Peak V̇O_2_; ml.kg^‐1^.min^‐1^	17.1 (4.0)	15.9 (2.8)	‐1.2	0.283	17.8 (1.6)	17.1 (1.9)	‐0.8	0.534	0.823
Work rate; W	98 (33)	102 (29)	4	0.419	105 (35)	116 (45)	12	0.034	0.309
Heart rate; bpm	118 (22)	112 (24)	‐6	0.633	113 (29)	129 (15)	17	0.040	0.082
Duration of test; s	612 (66)	613 (45)	1	0.972	622 (58)	653 (72)	31	0.023	0.219

Volumes are reported at body temperature, ambient pressure and gas saturated with water vapour (BTPS). Duration of test was the time to reach peak V̇O_2_. V̇_E_/V̇CO_2_, ventilatory equivalent for CO_2_.

**Figure 3:**
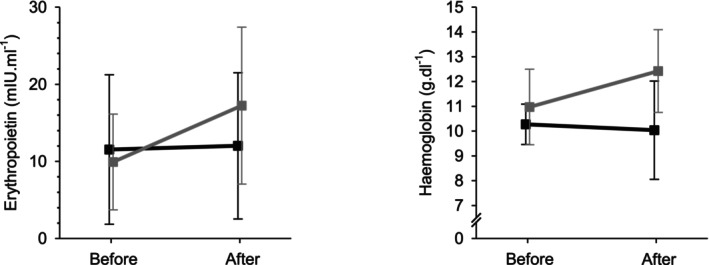
Haematological effects of residential hypoxia exposure. Erythropoietin and haemoglobin concentrations are shown before and after residential exposures. Black, normoxic week; grey, hypoxic week. Error bars indicate SD.

Both haemoglobin and erythropoietin significantly increased by the end of the hypoxic week compared with the normoxic week (Fig. 3 and Table 3). Haemoglobin was well‐matched across the exposures at baseline but was increased by (mean (SD)) 1.5 (2.7) g.dl^‐1^ (13% increase) after a week of hypoxia, while erythropoietin was increased by 74% at the end of the hypoxic week.

**Table 3:** Haematological data before, during and after exposure to normoxia and hypoxia. Values are mean (SD).

	Normoxia			Hypoxia			p value
	Day 0	Day 4	Day 8	Day 0	Day 4	Day 8	
Haemoglobin; g.dl^‐1^	10.3 (0.8)	11.8 (1.2)	10.0 (1.7)	11.0 (1.5)	11.3 (1.7)	12.4 (1.7)	0.028
Haematocrit; %	30 (2)	35 (3)	30 (6)	32 (5)	33.3 (5)	37 (5)	0.022
Erythropoietin; mIU.ml^‐1^	12 (10)	12 (7)	12 (9)	10 (6)	25 (21)	17 (10)	0.004

Day 0 data was obtained the day before commencing a week‐long exposure. Day 8 data was obtained immediately after completing a week‐long exposure.

## Discussion

Evidence supports the hypothesis that passive mild hypoxia, at a level sufficient to switch on hypoxia‐responsive genes and stimulate systemic responses, and of a duration short enough to be effective within the prehabilitation window, might achieve meaningful physiological benefits pre‐operatively that could translate into improved clinical outcomes postoperatively. This study began to explore the physiological potential for ‘altitude prehabilitation’ using a safe and practical ‘dose’ of mild hypoxia in older sedentary participants and found that one week of hypoxic conditioning had no effect on CPET variables that have been associated with peri‐operative outcomes. While there was an indication that participants were able to exercise longer and harder following a week of hypoxia, these changes were not statistically significant overall when compared with the control week. However, after a week of hypoxia, there was a large increase in erythropoietin in conjunction with a clinically significant increase in haemoglobin.

This study has established the technical feasibility of undertaking residential hypoxic conditioning in a context relevant to prehabilitation. In response to an F_I_O_2_ of 15%, the decrement in SpO_2_ during residential hypoxic conditioning was small and well tolerated but was nevertheless sufficient to activate classic hypoxia response mechanisms, consistent with responses seen during airline travel and on ascent to equivalent moderate altitudes. Building on extensive experience and research in these settings, the current study supports the use of 15% oxygen as an appropriate hypoxic stimulus for related peri‐operative research, although other F_I_O_2_ ‘doses’ may also be worthy of investigation.

With regards to the duration of simulated altitude exposure, the minimal time required to achieve meaningful gains is unknown for the population of interest. Elite young athletes may require two (or more) weeks of altitude exposure to achieve fitness benefits, but the underlying cellular and systemic physiological responses develop within hours of onset of hypoxia and much shorter periods may be sufficient in patients who are unfit. The relevant hypoxia physiology literature encompasses a very wide range of different altitudes, protocols, study populations and methodologies, and distilling this diverse and inconsistent evidence base into an optimal duration for older sedentary individuals is not straightforward. A duration of one week was carefully chosen as a sensible initial compromise between competing biological and logistical imperatives, but this period proved to be insufficient to achieve substantial improvements in CPET outputs. Two weeks may well have induced changes more consistent with those reported in the altitude training literature, but such a long period of residential hypoxia is unlikely to be practicable in the clinical setting. Studying a group with even lower baseline anaerobic threshold and peak V̇O_2_ may likewise have led to more pronounced changes, although the participants were objectively unfit and had raised BMI and body fat percentage, anaemia and impaired spirometry, in keeping with many patients.

It is possible that alternative hypoxic prehabilitation protocols would be more effective, such as intermittent exposure to short periods of hypoxia. Intermittent hypoxia has been used for therapeutic purposes for many years in former Soviet states, and available data in the Western literature supports its potential in a range of conditions [25]. For example, in a study of 30 sedentary volunteers, a total dose of 20 h of 15% oxygen (four weeks of 1‐h exposures) was found to increase anaerobic threshold by 20% and V̇O_2_max by 10% [32]. However, results vary widely across the literature and robust clinical validation is still required. Overnight exposure is another alternative that warrants consideration. Drawing from the popular ‘sleep high, train low’ regimen, a home‐based approach using a hypoxic canopy or tent while sleeping may offer distinct practical advantages and has attracted interest in other areas of medicine (e.g. as a future treatment for type‐2 diabetes).

A further possibility could be to use hypoxia and exercise in an integrated programme. Although conclusive data have proven elusive and current evidence is relatively weak, there is widespread acceptance that exercise prehabilitation probably does improve physical capacity before surgery and reduce postoperative complications [3, 4, 5, 6]. The addition of intermittent hypoxia may augment any potential benefits of an exercise programme, including classical cardiopulmonary function but also encompassing cellular mechanisms influencing metabolic flexibility, antioxidant capacity, glycaemic control, endothelial regeneration and anti‐inflammatory processes.

Although simulated altitude exposure did not substantially affect CPET‐derived variables, this study has notably introduced haematological fitness as a distinct route for hypoxia‐induced physiological risk modification that goes beyond the mediating factors linked to exercise and hypoxia above. Pre‐operative anaemia is common, affecting more than 30% of patients undergoing major surgery and is a poor prognostic indicator [7, 8, 9]. It is associated with an increase in postoperative complications including mortality [7, 8, 9], and peri‐operative blood transfusions also worsen outcomes [10]. Pre‐emptive correction of low haemoglobin is consequently a priority in modern peri‐operative care and the first pillar of patient blood management, with iron therapy (oral or intravenous) as the mainstay [7]. There is broad scope for improving pre‐operative management [7, 10] and while correcting anaemia is the essential aim, we note that even elite endurance athletes achieve benefits from ‘blood doping’ [33]; non‐anaemic patients may benefit from boosting haemoglobin mass even within the normal range as part of a continuum of optimisation [8, 33]. As noted by others, comparing performance gains in elite athletes to patients undergoing surgery involves “*a substantial conceptual leap*” [33] and hypoxic conditioning currently falls outside mainstream clinical experience. Nevertheless, there is a sound scientific rationale for this means of improving patients' physiological reserve, and if the 1.5 g.dl^‐1^ increase in haemoglobin induced by simulated altitude in this study were translated to the clinical setting, it would be considered highly likely to improve patient outcomes [9].

Alongside advances in intra‐operative and postoperative care [34], there is an urgent need to examine all potential means of modifying risk pre‐operatively. ‘Altitude prehabilitation’ is a novel concept, and the current study was intended to provide an initial physiological foundation using a conventional physiology study design and, through this, establish a basis for further investigation. Although it is possible that the sample size limited the ability to detect subtle changes, our aim was to identify key physiological phenomena rather than pursue minor or esoteric effects. Overall, the results suggest there is indeed value in exploring hypoxic conditioning which, like all other modalities of prehabilitation, may not necessarily revolutionise clinical outcomes in isolation, but might nevertheless make a valuable contribution to cumulative ‘marginal gains’ in combination with other interventions.

In summary, this study has established the concept and feasibility of using simulated altitude exposure as a means of prehabilitation and has demonstrated that one week of mild hypoxic conditioning can induce potentially beneficial physiological changes, although key CPET variables were not affected. Optimising haematological fitness is a logical focus for future studies of ‘altitude prehabilitation’, which are required to determine whether this form of physiological manipulation can meaningfully contribute to reducing peri‐operative morbidity and mortality.
